# Description of immature stages and biological notes of *Cassidisparelicta* Medvedev, 1957, a newly recorded species from China (Coleoptera, Chrysomelidae, Cassidinae, Hispini)

**DOI:** 10.3897/zookeys.780.23280

**Published:** 2018-08-08

**Authors:** Chengqing Liao, Zhilin Zhang, Jiasheng Xu, Charles L. Staines, Xiaohua Dai

**Affiliations:** 1 Leafminer Group, School of Life and Environmental Sciences, Gannan Normal University, Ganzhou, Jiangxi 341000, China Gannan Normal University Ganzhou China; 2 Ulanqab Municipal Forestry Pest Control and Quarantine Station, Ulanqab, Inner Mongolia 012000, China Ulanqab Municipal Forestry Pest Control and Quarantine Station Ulanqab China; 3 Department of Entomology, National Museum of Natural History, Smithsonian Institution, P. O. Box 37012, Washington, DC 20013-7012, USA National Museum of Natural History, Smithsonian Institution Washington United States of America; 4 National Navel-Orange Engineering Research Center, Ganzhou, Jiangxi 341000, China National Navel-Orange Engineering Research Center Ganzhou China

**Keywords:** Cassidinae, *
Cassidispa
*, chaetotaxy, Hispini, immature stages, leaf-mining insects, morphology

## Abstract

The first instar and mature larva and pupa of *Cassidisparelicta* Medvedev, 1957, a newly recorded species from China, are described and figured. The chaetotaxy of the head, mouthparts, legs, and dorsal and ventral surfaces of the body is described. This is the first detailed description of immatures in the genus *Cassidispa*. Diagnostic characters of this species are compared with other described immatures of some Hispini genera. Biological notes on *C.relicta*, such as host plants, feeding patterns of adults, structure of larval mines and life history, are also presented.

## Introduction

*Cassidispa* Gestro, 1899 is a leaf beetle genus belonging to the tribe Hispini Gyllenhal, 1813 (Chrysomelidae: Cassidinae), with eight species occurring in China, Russia, Angola, Democratic Republic of Congo and Zimbabwe (Staines 2015). Four species are previously recorded from China: *C.bipuncticollis* Chen, 1941, *C.femoralis* Chen & Yu, 1976, *C.maderi* Uhmann, 1938 and *C.mirabilis* Gestro, 1899 (Chen et al. 1986; T’an 1993; Hua 2002; Staines 2015). Adults of the genus *Cassidispa* can be distinguished from all other genera of Hispini by the anterior margin of the pronotum without spines, by the pronotum and elytra with broadly expanded margins, and by the antennae having nine antennomeres (Chen et al. 1986). The main diagnostic characters between *Cassidispa* and the similar genus *Platypria* Guérin-Méneville, 1840 are the lateral margins of pronotum expanded from base to anterior angle with irregular translucent patches, the continuous expanded margins of the elytra, the elytra with very low and obtuse tubercles, never with spines, and the lateral margins of elytra with numerous small spines not with long ones (Chen et al. 1986).

There is little published biological information on *Cassidispa* species. Hua (2002) listed the host of *C.bipuncticollis* as *Betula* spp. (Betulaceae) from China. Recently, we discovered that the larvae of *C.relicta* mine in the leaves of *Betulaplatyphylla* Suk. (Betulaceae) and *Ulmuspumila* Linn. (Ulmaceae) in Inner Mongolia, China. The species reached outbreak levels in 2016–2017 and became a potential pest of the dominant trees in the area. As Dr. Lukáš Sekerka pointed out, the species is *Cassidisparelicta* Medvedev, 1957 not *C.mirabilis*. Both species are superficially similar by predominantly black coloration but they are distinct: *C.relicta* has generally strongly shiny dorsum (but not as shiny in *C.mirabilis*); *C.relicta* has shorter and thicker antennae, explanate margin of pronotum largely black (but yellow in *C.mirabilis*); *C.relicta* has anterior spots on explanate margin of elytra almost reaching to humeri (but widely separated from humeri in *C.mirabilis*); elytra of *C.relicta* is very distinctly constricted in 3/4 length (but weakly in *C.mirabilis*) (Lukáš Sekerka, Personal Communications). *C.relicta* is previously reported in Russia (Medvedev 1957; Staines 2015). Therefore, it is a newly recorded species from China and China hosts all five Asian *Cassidispa* species now.

In this publication, we describe the larvae and pupa of *C.relicta* and provide the first detailed report on immature morphology and biological information for the genus *Cassidispa*.

## Materials and methods

**Figures 1–3.**
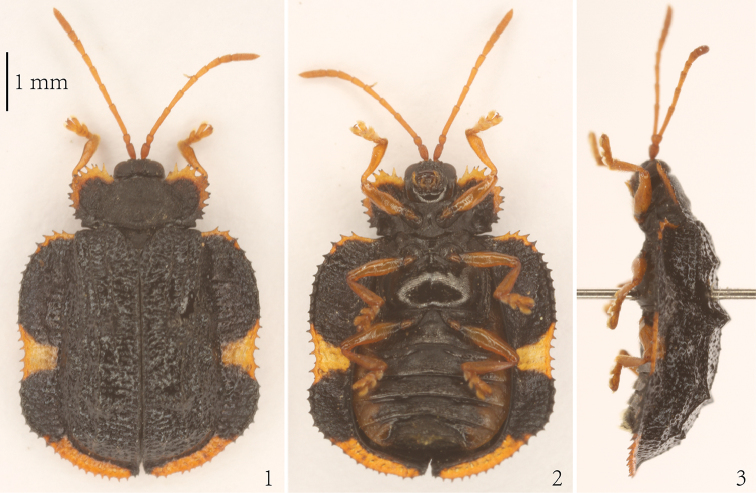
*Cassidisparelicta*. **1** Dorsal view **2** Ventral view **3** Lateral view.

All immatures and adults were observed and collected at Shanggaotai Forest Farm (Zhuozi County, Inner Mongolia) from March 2016 to October 2017. Immatures and adults of *C.relicta* were collected on wild plants and some of them were preserved in anhydrous ethanol. Some adults were pinned in the laboratory (Figs 1–3) and determined using the keys in Chen et al. (1986). Four first-instar larvae, three mature larvae and three pupae were examined morphologically. For microscopic study, the heads of the larvae were separated from the rest of the body, boiled in 10% NaOH solution and cleaned in water before dissecting the mouthparts.

The photos of adults were taken using a Cannon EOS 7D camera with macro lenses; the dissection of heads and mouthparts was made with a Motic SMZ-140 and Olympus SZX2-ILLT stereomicroscope; figures and examination were performed using an Optika B-292 microscope and Cannon EOS 70D camera. Our descriptions of immature stages follow Świętojańska et al. (2006). The terminology of the chaetotaxy of the head follows Borowiec and Świętojańska (2003). All studied material (first-instar larvae, mature larvae, and pupae) and adults were deposited at the Leafminer Group, School of Life and Environmental Sciences, Gannan Normal University (Ganzhou, China).

## Results

### Cassidispa relicta

Taxon classificationAnimaliaColeopteraChrysomelidae

Medvedev, 1957

#### Larva

(Figs 4–17). Length of mature larva 6.1–6.2 mm without head, width 1.6–1.7 mm across pronotum. Length of first instar larva 1.4–1.8 mm without head, width of body 0.7–0.8 mm across pronotum.

Body distinctly flattened dorso-ventrally. Pronotum of first instar larvae slightly wider than abdominal segments; mature larvae widest across abdominal segments IV–V (Figs 6–7, 16–17). Body color of alcohol-preserved larvae yellowish-white with two irregular brown patches on pronotum (paler and without dark markings in first instar larvae), black anterior margin of abdominal segment IX, dark brown spiracles, yellowish-brown triangular patch on prosternum, brown head and legs. Abdomen of live larvae dark brown or black (Figs 31–32).

**Figures 4–5.**
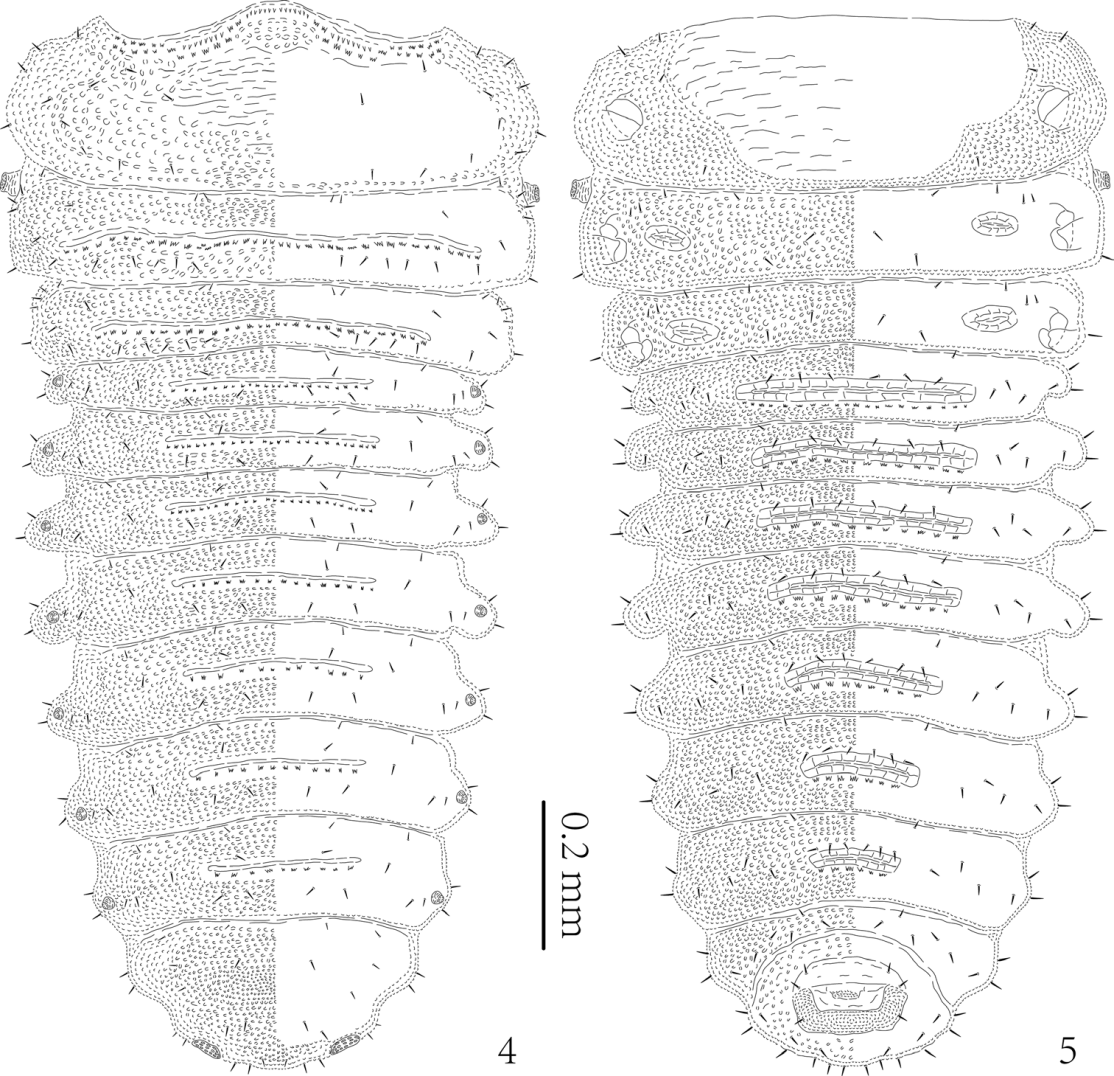
*Cassidisparelicta*, first instar larva. **4** Dorsal view **5** Ventral view.

**Figure 6.**
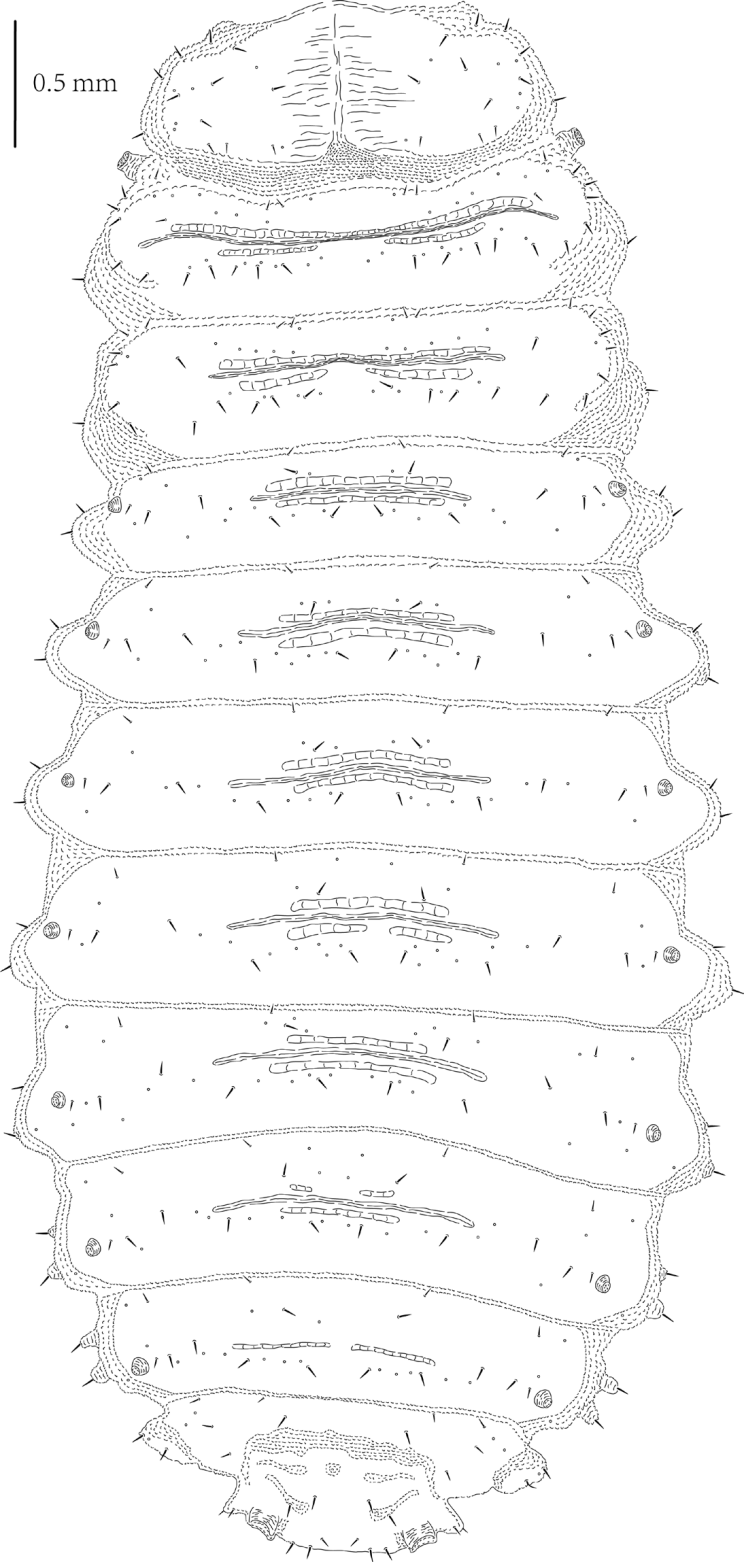
*Cassidisparelicta*, last instar larva, dorsal view.

**Figure 7.**
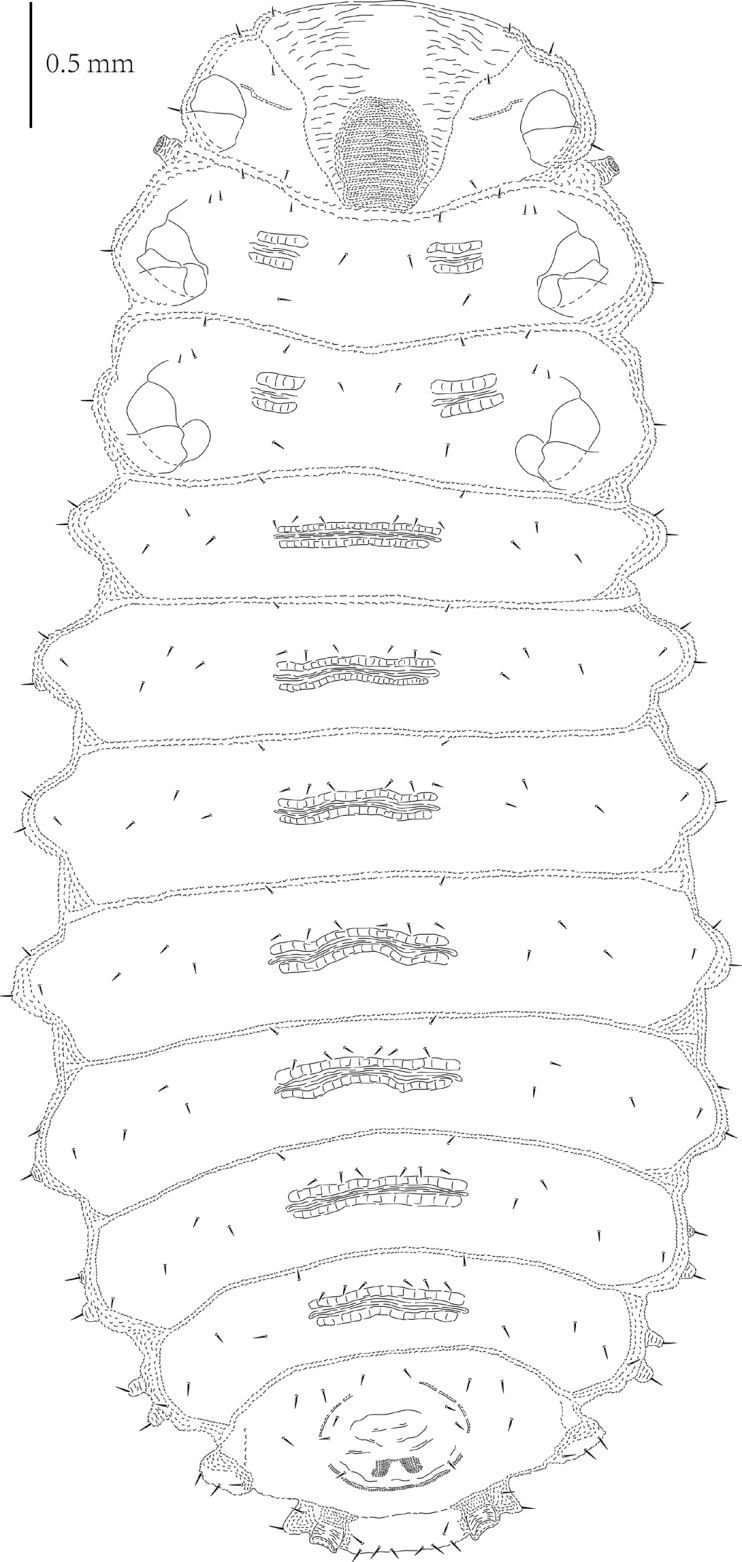
*Cassidisparelicta*, last instar larva, ventral view.

Body with eight pairs of lateral scoli on abdominal segments (Figs 6–7, 16–17). Lateral scoli short and round, approximately of same length; scoli of segments VI–VII with two small simple lateral branches (first instar larvae without lateral branches as in Figs 4–5). All lateral scoli with two setae apically and one seta ventrally.

Granulation of body distinct in all examined specimens including both first instar and mature larvae. Each tergite and sternite (except for sternite VIII) with minute setae on anterior margin; tergites and sternites covered with short pointed setae. Tergites of meso- and metathorax, abdominal segments I–VI and sternites I–VII of abdomen with transverse grooves (Figs 4–7). Sternites of meso- and metathorax with two short transverse grooves medially, very similar in shape to other tergites and sternites. Posterior margin of each transverse groove and anterior margin of pronotum with distinct asperites.

Pronotum with four setae on each lateral margin, five setae on each lateral side, and three setae close to posterior margin (Figs 4, 6). Meso- and metanotum with six minute setae on anterior margin: two pairs at middle and one pair laterally; row of ten setae running across segment; group of six setae laterally. Meso- and metanotum with one seta on slightly visible protuberance laterally. Abdominal tergites I–VII with four setae on anterior margin; two rows of setae running across segment: anterior with two setae, posterior with four setae; three setae close to each spiracle (seta closest to spiracle minute). Abdominal tergite VIII with four minute setae on anterior margin; three rows of setae running across segment: anterior with four setae, median with two setae, and posterior with four setae (between spiracles). Posterior margin of abdominal segment VIII with ten setae: two pairs close to spiracles laterally, three pairs between spiracles medially.

Prosternum with one seta in each anterior angle; one seta laterally at base of leg; and two setae close to posterior margin (Figs 5, 7). Meso- and metasternum with four setae on anterior margin; two rows of setae running across segment medially, both with two setae; and two setae laterally at base of leg. Abdominal sternites I–VII with pair of minute setae on anterior margin medially; with row of six setae running across segment medially; and three setae laterally. Abdominal sternite VIII with row of eight setae across segment anteriorly and two setae posteriorly; with two setae close to each spiracle; four setae along anterior base of anus.

Nine pairs of distinct spiracles (Figs 4, 6, 16): one on thorax and eight on abdomen. Thoracic spiracles distinctly more elevated than abdominal spiracles, diameter of spiracles of abdominal segments I–VII approximately equal, but spiracles of abdominal segment VIII distinctly larger, flattened, slightly elevated.

**Figures 8–15.**
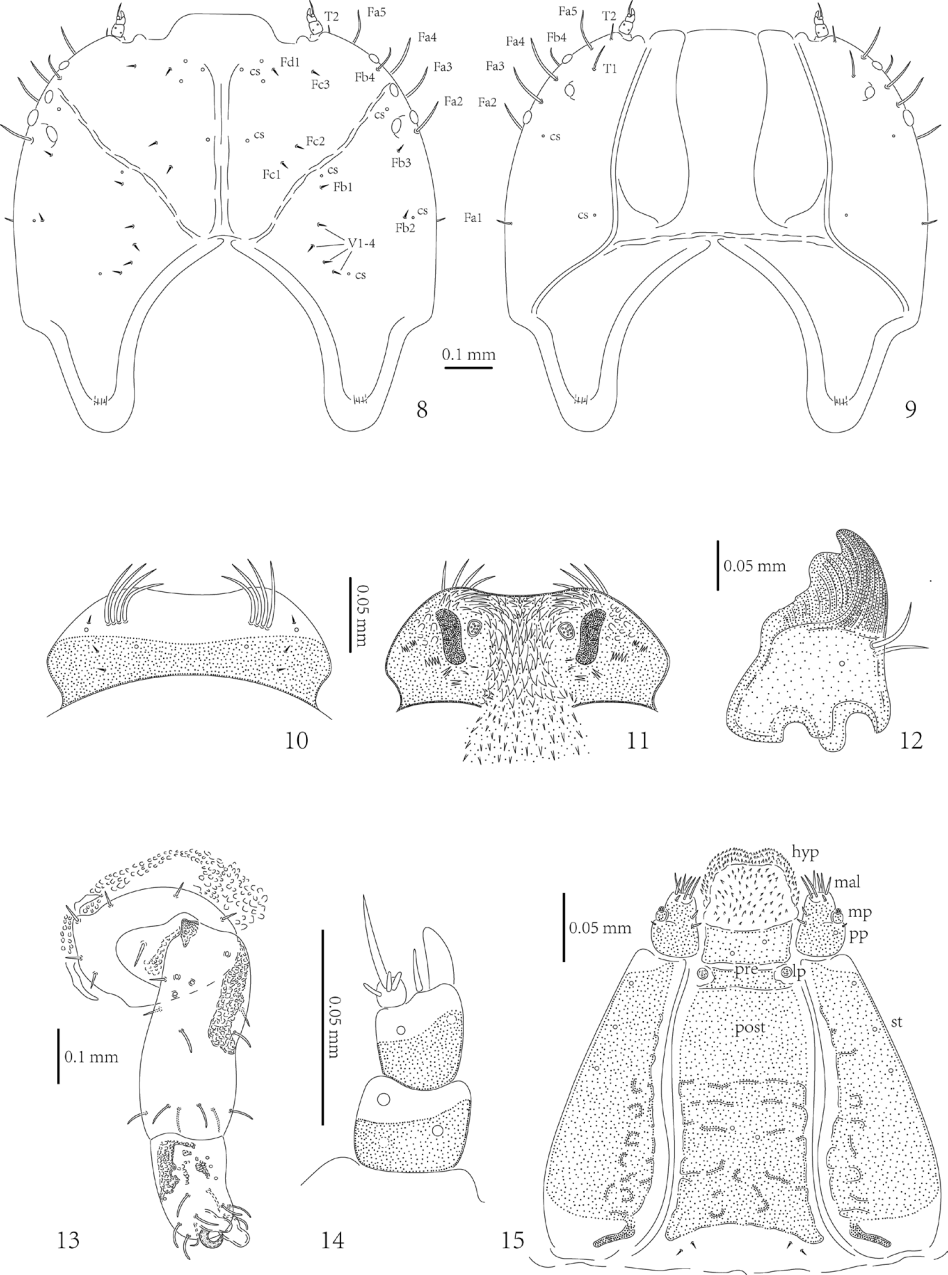
*Cassidisparelicta*, larva. **8** Dorsal view of head: cs – campaniform sensilla **9** Ventral view of head **10** Dorsal view of labrum **11** Ventral view of labrum **12** Mandible **13** Leg **14** Antenna **15** Maxillae and labium, ventral. Abbreviations: hyp – hypopharynx; mal – mala; mp – maxillary palp; pp – palpifer; lp – labial palp; pre – prementum; post – postmentum; st – stipes.

Head well sclerotized, prognathous, partially retracted into pronotum (Figs 8–9). Epicranial stem absent; median endocarina wide, extending between frontal arms; frontal arms V-shaped, fronto-clypeal suture absent. Clypeus wider than long, without setae and campaniform sensilla. Frons with two short setae (Fd1 and Fc3) and three campaniform sensilla laterally, two setae (Fc1 and Fc2) and one campaniform sensillum between median endocarina and frontal arm, one long seta (Fb4) laterally close to frontal arm; vertex with seven short setae (Fb1, Fb2, Fb3, and V1-4) and four campaniform sensilla (three respectively close to Fb1, Fb2 and V4, one between Fb4 and dorsal stemmata). One long seta (Fa1) on lateral margin close to pronotum, four long pointed setae (Fa2, Fa3, Fa4, and Fa5) close to stemmata. Temporal side with two long setae (T2 close to antenna, and T1 between T2 and ventral stemmata) and two campaniform sensilla.

Five stemmata laterally: four dorsal-laterally, one ventrally (Figs 8–9). Antenna with three antennomeres, set in membranous ring (Figure 14). First antennomere stout, approximately as wide as long, with three campaniform sensilla; second antennomere stout, slightly longer than wide, with two campaniform sensilla, one prominent sensory appendix apically, and one stout seta close to third antennomere; third antennomere very short, with long, pointed seta and three peg-like sensilla.

Labrum approximately three times wider than long, anterior margin slightly emarginate (Figs 10–11), dorsal surface with: three setae and one campaniform sensillum laterally; one pair of campaniform sensilla medially; and four stout setae laterally close to anterior margin. Mid- and anterior areas of ventral surface with numerous stout spines; lateral and posterior areas with tiny spines; two irregular groups of few small sensilla medially.

Mandibles heavily sclerotized, with two prominent teeth (Figure 12): anterior distinct and conical, posterior blunt; followed by some tiny teeth. Two long setae very close to each other and one campaniform sensillum.

Maxillae and labium connate (Figure 15). Each stipes (st) with three campaniform sensilla anteriorly. Palpifer (pp) with one small seta apically and one campaniform sensillum laterally. Maxillary palp (mp) with two palpomeres: first palpomere stout, second palpomere with group of small peg-like sensilla at apex. Mala (mal) with six long pointed setae and one short seta apically, and two setae subapically. Hypopharynx (hyp) covered with numerous small spines. Labial palp (lp) with one palpomere, with group of small peg-like sensilla at apex. Prementum (pre) with two campaniform sensilla anteriorly. Postmentum (post) with two pairs of short setae at base and two campaniform sensilla medially.

Legs oblong, consisting of three segments: coxa, femur, and tibiotarsus (Figure 13). Tibiotarsus armed apically with claw. Coxa with four setae along base on internal surface, and one seta dorsally and two setae on external surface. Femur with five setae and four campaniform sensilla on basal half, and six long setae on apical half. Tibiotarsus with nine long pointed setae and two campaniform sensilla: two setae at middle laterally, six setae around claw apically, one seta and two sensilla above claw. Base of claw with distinct pulvilli.

#### Pupa

(Figs 18–21). Length of pupa 5.2 mm, width 1.7 mm across base of pronotum and 2.5 mm across abdominal segment IV without lateral scoli.

Body flattened dorso-ventrally, elongate-oval. Color of live as well as alcohol-preserved pupa brownish yellow, mesothorax with two dark markings at base of wings, apex of abdomen dark brown (Figs 18–19).

Body, especially abdomen, distinctly granulate (Figs 18–21). Abdominal tergites I–III with two short transverse grooves medially. Abdominal tergites IV–VII each with long transverse groove. Tergites of abdominal segments I–VII with several small tubercles around grooves. Tubercles of tergites VI–VII arranged as row close to posterior margin. Sternites of abdominal segments IV–VII each with two transverse ridges close to posterior margin.

Head visible in dorsal view (Figs 18, 20). Prothorax trapezoidal in shape. Thorax without lateral scoli. First abdominal segment without lateral scoli. Segments II–V with single simple scolus laterally and small low tubercle anteriorly armed with pointed seta. Abdominal segment VI with two lateral scoli. Segments VII–VIII each with three lateral scoli. Each scolus apically armed with one seta. Posterior lateral scolus of segments VI–VIII with one lateral branch directed posteriorly without setae. Segment VIII additionally with two broad, flattened processes on posterior margin; each with six setae apically.

Head with three pairs of setae laterally and four setae anteriorly (Figure 20). Pronotum with group of nine setae laterally. Mesonotum with three pairs of setae medially. Metanotum with oblique row of four setae on each side medially. Abdominal tergites I–VII with two rows of setae running across segment: anterior with two setae, posterior with four setae; two setae close to each spiracle. Abdominal tergite VIII with three rows of setae running across segment: anterior with four setae, median with four setae, and posterior with two setae medially; one seta very close to each spiracle.

In ventral view (Figure 21), head with group of 14 setae: two setae anteriorly, four setae medially, six setae posteriorly at base of labrum, and two setae at base of mandibles. Tarsus of leg apically armed with one pointed seta. Visible abdominal sternites IV–VII with four setae on anterior margin, row of six setae medially, two setae laterally, and one seta at base of lateral scoli ventrally. Abdominal sternite VIII with rows of six setae anteriorly and six setae along anterior base of anus.

Abdominal segments each with one pair of spiracles (Figure 21). Spiracles of segment I smaller than others, spiracles of segments II–IV and VI–VII similar in shape but approximately twice as large as spiracles of segment I. Spiracles of segment VIII not elevated, oblong. Spiracles of segment V prominent, elongated into cylindrical appendage (respiratory horns), directed posteriorly.

#### Biological notes.

The biology of the genus *Cassidispa* is poorly known. There is only one species having confirmed host plant record. Hua (2002) reported *C.bipuncticollis* to be associated with *Betula* (Betulaceae), but it is just a list even without some evidences. Our rearing of *C.relicta* on *Betula* and some other trees indicates that there might be more larval host associations for the genus. Additionally, the immature stages of the genus *Cassidispa* were completely unknown and this paper was the first study about it.

The larvae of *C.relicta* were found mainly mining in the leaves of *Betulaplatyphylla* (Figs 22–25, 27–34) and occasionally mining the leaves of some other trees, such as *Ulmuspumila* Linn. (Ulmaceae) (Figure 26), *Populusdavidiana* Dode, *P.cathayana* Rehd. (Salicaceae) and *Armeniacasibirica* (L.) Lam. (Rosaceae).

The life cycle of *C.relicta* is univoltine based on our field observations throughout 2016–2017. *C.relicta* overwinters as a mature larva until the temperature rises and the soil thaws in the spring. The mature larvae break dormancy and pupate in the fallen leaves in early April (Figs 33–34). The pupal stage lasts about one month. Adults emerge in late May (the freshly emerged adults are mostly white, with antenna and legs brown, and disc of pronotum black) and feed on the mesophyllic tissue of the upper surface of host leaves (Figs 22–24). The feeding pattern of the adults is usually irregular and may densely cover the leaves (Figs 22–25, 28–32). In early June, the adults start to copulate (Figs 23–24), and fertilized females lay an egg within a shallow hole which was chewed on the lower leaf surface, and then covered with feces (Figs 25–26). Adults have no apparent feeding preferences for young or old leaves, but females generally do not oviposit on new leaves. Females oviposit eggs scatteredly in a one-by-one way (Figs 24–26, 28). The newly deposited eggs are usually milk-white and translucent and its covered feces turn brown over time (Figs 25–26, 28). Sometimes, females will lay some eggs on the tree trunk, perhaps when they could not find any suitable leaves (Figure 27). The egg stage lasts about 20 days. A freshly hatched larva bores into the mesophyllic tissue and forms a large irregular mine with a resting place (dark color) surrounding the oviposition point (Figure 29). The larvae deposit their feces in their own mine (Figs 29–32). Each younger larva has its own absolute mine at the early stage which will combine with other mines on the same leaf over time (Figs 29–32). One leaf usually has three larvae or, in an outbreak, up to seven larvae. If one leaf does not likely provide enough food for these larvae to complete their development, they leave their original mine, migrate to a new leaf, and construct a new mine (Figs 31–32). The final larval mine is a large irregular blotch type, almost without any mesophyllic tissue left (Figs 30–32). The mined leaves gradually become yellow and may dry-up or abscise early. In early October the mature larvae leave their mine to enter a fresh uneaten area of the same leaf to construct a pupal mine (Figs 33–34). The mature larvae of *C.relicta* does not directly go into pupation like other leaf-mining hispines (Świętojańska and Kovac 2007; Lee et al. 2009; Liao et al. 2014; Liao et al. 2018) but into a long dormant period for overwintering. However, it is a shortcoming we did not perform the larval instar observations.

## Discussion

General morphology of the larva and pupa of *C.relicta* is typical for species of leaf-mining Hispini. The immature stages of Hispini beetles have been reported on some species, such as *Acmenychusinermis* (Zubkoff, 1833) (Medvedev 1968), *Dactylispasetifera* (Chapuis, 1877) (Chen et al. 1986), *D.xanthopus* (Gestro, 1898) (as *D.chinensis* Weise, 1905), *D.doriae* Gestro, 1890, *D.chaturanga* Maulik, 1919, *D.xanthospila* Gestro, 1890 (Zaitsev 2012), *D.ignorata* Uhmann, 1953 (as *D.chapuisi* (Gestro, 1890)), *D.rufiventris* (Kraatz, 1895) (Maulik 1932), *D.feae* (Gestro, 1888) (as *D.flavomaculata* Uhmann, 1930), *D.issiki* Chûjô, 1938 (Fukuda and Kurosa 1959), *D.higoniae* (Lewis, 1896), *D.subquadrata* (Baly, 1874) (Yano 1965), D. hystrix (Duvivier, 1891) (Paulian 1949), *D.cladophora* (Guérin-Méneville, 1841), *D.nemoralis* (Gestro, 1897), *D.vethi* (Gestro, 1906) (Uhmann 1956), *D.callosa* Uhmann, 1935 (Uhmann 1962), *Dicladispaarmigera* (Olivier, 1808) (Chen et al. 1986; Kimoto and Takizawa 1994; Lee and Cheng 2010), *Dicladispatestacea* (Linnaeus, 1767) (Świętojańska et al. 2014), *Hispaatra* Linnaeus, 1767 (Grandi 1935), and *Platypriaerinaceus* (Fabricius, 1801) (as *P.andrewesi* Weise, 1904) (Uhmann 1957), *P.kapauku* Gressitt, 1957 (as *P.linnei* Weise, 1905) (Gressitt 1963), *P.melli* Uhmann, 1954 (Kimoto et al. 1997; Liao et al. 2014) and so on. However, some early literatures had no detailed information or illustrations on their immature stages. In this paper, some diagnostic characters of the immature stages among several representative species in the genera of *Cassidispa*, *Dactylispa*, *Dicladispa* and *Platypria* are compared and summarized in Table 1.

**Table 1.** Comparisons of diagnostic characters of immature stages among *C.relicta* and some species in the genera of *Dactylispa*, *Dicladispa*, and *Platypria*.

Diagnostic characters	Larva			Pupa			References
	Lateral scoli on meso- and metathorax	Shape of abdominal scoli	Lateral branches of abdominal scoli	Processes on pronotum	Processes on abdominal apex	Spiracles of fifth abdominal segment	
* C. relicta *	absent	rounded	segments VI–VII	absent	2 flattened	short, thick	This paper
* D. setifera *	absent	triangular	absent	absent	2 flattened	short, pointed	Chen et al. 1986
* D. rufiventris *	absent	diminutive	absent	absent	2 spinulose	short, pointed	Maulik 1932
* D. chapuisi *	absent	rounded	absent	absent	2 spinulose	spiniform	Maulik 1932
* D. javaensis *	present	triangular	absent	absent	2 small	long conical	Maulik 1931
* D. higoniae *	present	triangular	absent	absent	2 small triangular	long conical	Yano 1965; Kimoto et al. 1997 (larva); Lee and Cheng 2007
* D. insulicola *	present	triangular	absent	absent	2 flattened	long conical	Lee and Cheng 2007
* D. latipennis *	present	triangular	absent	absent	2 flattened	long conical	Lee and Cheng 2010
* D. doriae *	present	triangular	absent	absent	2 pointed	upward-hooked	Zaitsev 2012
* D. xanthopus *	present	triangular	absent	absent	2 short	upward-hooked	Zaitsev 2012
* D. haturanga *	present	triangular	segments VI–VII	absent	2 branched	long conical	Zaitsev 2012
* Di. armigera *	absent	triangular	absent	present	6 small spinous	long pointed	Chen et al. 1986; Kimoto et al. 1997 (larva); Lee and Cheng 2010
* Di. testacea *	absent	short, fine	absent	absent	2 flattened	elongate-horned	Świętojańska et al. 2014
* P. andrewesi *	present	triangular	absent	present	2 flattened	long conical	Uhmann 1957 (pupa); Kimoto et al. 1997 (larva)
* P. melli *	present	triangular	absent	present	2 flattened	long conical	Kimoto et al. 1997 (larva); Liao et al. 2014

**Figures 16–19.**
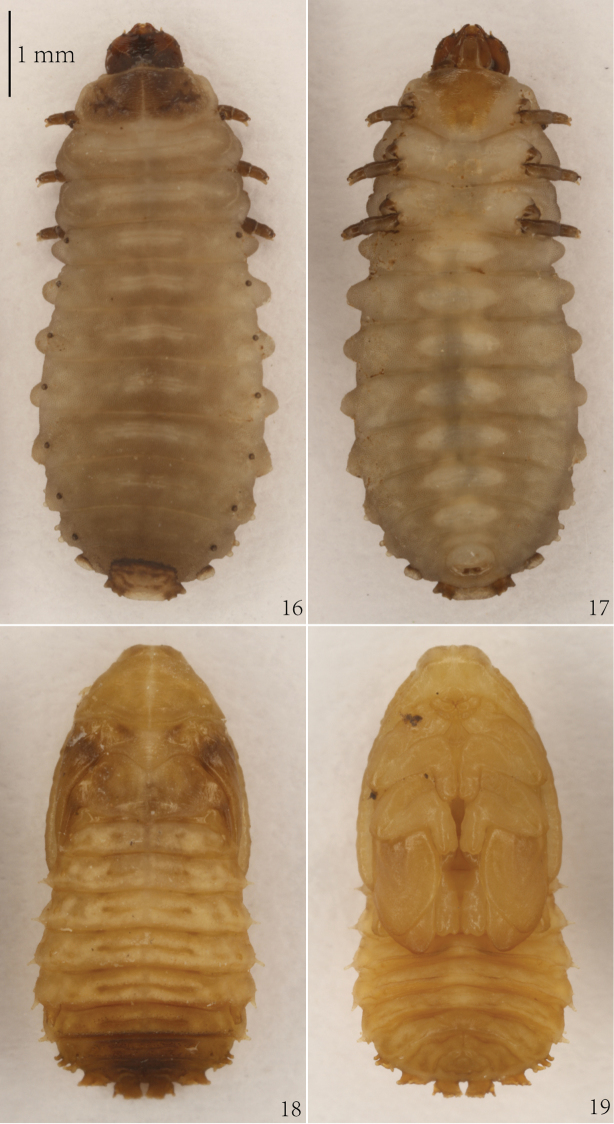
*Cassidisparelicta*. **16** Larva, dorsal view **17** Larva, ventral view **18** Pupa, dorsal view **19** Pupa, ventral view.

The larva of *C.relicta* is very similar to that of some *Dactylispa* species and can be distinguished by the lateral branches of abdominal scoli on segments VI–VII (very small and with a rounded apex), although this character also presented on *D.haturanga* but distinctly slender and with a pointed apex (Zaitsev 2012). Additionally, the abdominal processes are distinctly round in shape (Figs 6–7, 16–17), with a pair of spiracles whose opening directed backward (in genus *Dactylispa* the spiracles usually formed very pointed or triangular respiratory horns). The pupa of *C.relicta* is most similar to that of *Di.testacea*. These two species each have a pair of lateral scoli on abdominal segments I–V and have 2–3 pairs of lateral scoli on segments VI–VIII, the abdominal apex has two flattened processes; and the spiracles of the fifth abdominal segment look very similar. The differences are that the body shape of *C.relicta* is broadly ovate and the anterior margin of the head is slightly straight, but the head of *Di.testacea* is distinctly emarginate.

The larvae, especially younger larvae, usually returned to the resting place (Figure 29). Such an interesting and specific behavior was also found in some species of the genus *Dactylispa*, such as *D.subquadrata* (Baly, 1874) and *D.higoniae* (Lewis, 1896) (Yano 1965), but not in *D.angulosa* (Solsky, 1871) (Yano 1965) and *D.approximata* Gressitt, 1939 (Dai et al. 2012). The leaf areas with obviously deeper color smeared with larval feces which may help the larvae to avoid predators and parasitoids (Hering 1951; Yano 1965).

**Figures 20–21.**
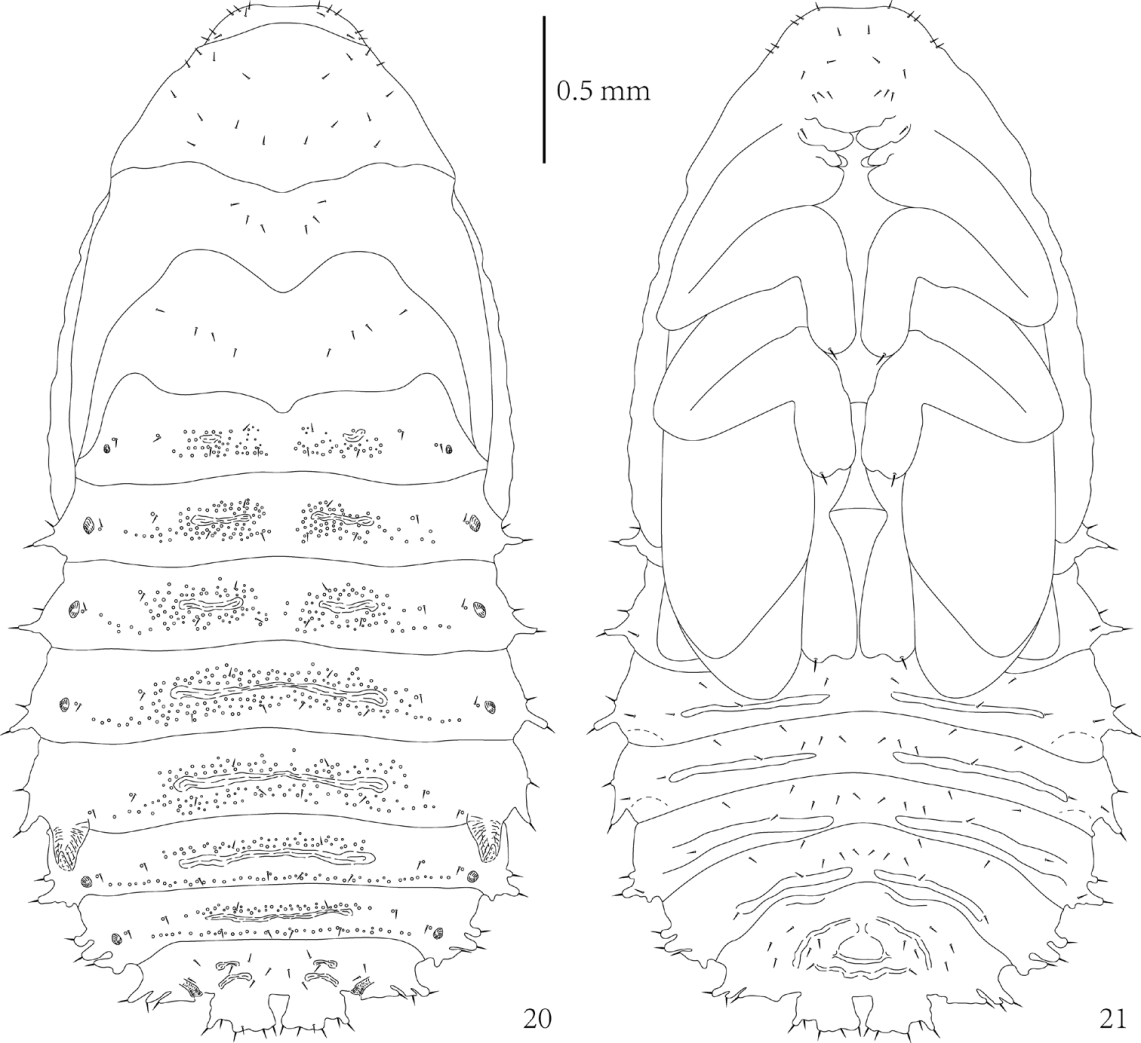
*Cassidisparelicta*, pupa. **20** Dorsal view **21** Ventral view.

**Figures 22﻿–27.**
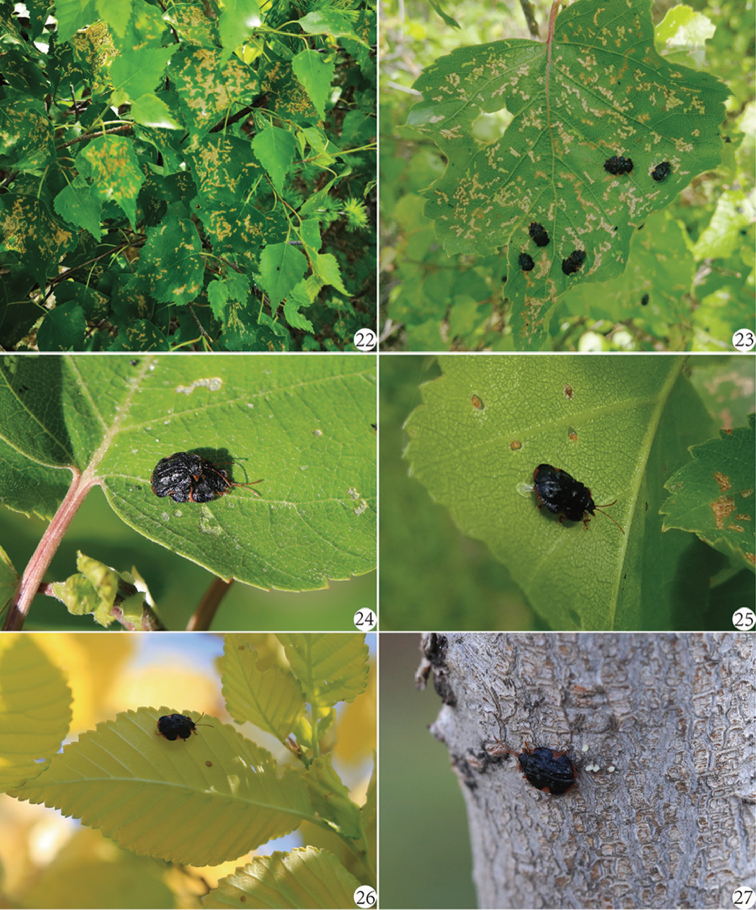
Life stages of *Cassidisparelicta* on host plants (all on *Betulaplatyphylla* except Fig. 26 on *Ulmuspumila*). **22–23** Adults and their feeding pattern **24** Adults copulate on upper surface of host leaf **25–26** Females laying eggs on lower surface of host leaves **27** Female laying eggs on the tree trunk.

**Figures 28–34.**
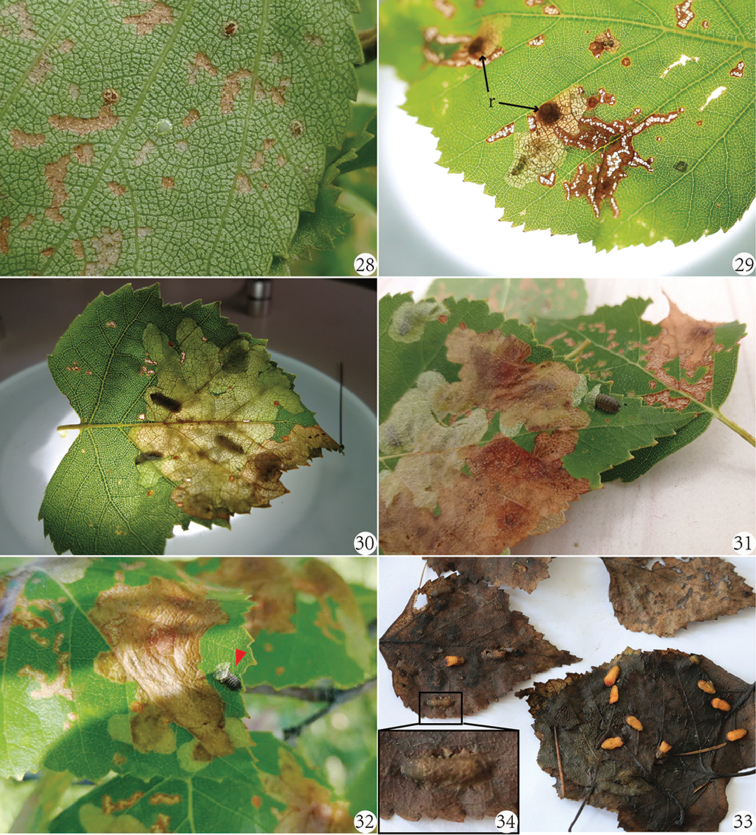
Life stages of *Cassidisparelicta* on host plants *Betulaplatyphylla*. **28** Eggs **29** A leaf with new mines of hatching larvae and larval resting places focusing on their egg points: r – resting place of larva **30** Shared large mine of three older larvae in a same leaf **31–32** Mature larva leaves its original mine and builds a new one for overwintering and pupation, in Fig. 32 the red arrow indicates the location of mature larva **33** Pupae in the leaf litter in spring **34** A pupal mine in the fallen leaf.

## Supplementary Material

XML Treatment for Cassidispa relicta

## References

[B1] Borowiec L Świętojańska J (2003) The first instar larva of *Cassidanebulosa* L. (Coleoptera: Chrysomelidae: Cassidinae) – a model description. Annales Zoologici 53: 189–200.

[B2] Chen SH Yu PY Sun CH T’an CH Zia Y (1986) Fauna Sinica (Insecta: Coleoptera: Hispidae). Science Press, Beijing, 653 pp. [In Chinese]

[B3] Dai X Xu J Jiang Z (2012) Bionomics of *Dactylispaapproximata* on *Lophatherumgracile*. Northern Horticulture 22: 125–127. [In Chinese]

[B4] Gressitt JL (1963) Hispine beetles (Chrysomelidae) from New Guinea. Pacific Insects 5(3): 591–714.

[B5] Hering EM (1951) Biology of leaf miners. Springer-Science + Business Media, Berlin, 420 pp 10.1007/978-94-015-7196-8

[B6] Hua LZ (2002) List of Chinese Insects Vol. 2. Sun Yat-sen University Press, Guangzhou, 612 pp.

[B7] Kimoto S Takizawa H (1994) Leaf beetles (Chrysomelidae) of Japan. Tokai University Press, Tokyo, 539 pp.

[B8] Lee CF Cheng HT (2010) The Chrysomelidae of Taiwan 2. Sishow–Hills, Taipei, 191 pp. [In Chinese]

[B9] Lee CF Świętojańska J Staines CL (2009) *Prionispahoujayi* (Coleoptera: Chrysomelidae: Cassidinae: Oncocephalini), a newly recorded genus and new species from Taiwan, with a description of its immature stages and notes on its bionomy. Zoological Studies 51: 832–861.

[B10] Liao C Xu J Dai X Zhao X (2014) Study on the biological characteristics of *Platypriamelli*. Northern Horticulture 3: 118–120. [In Chinese]

[B11] Liao C Liu P Xu J Staines CL Dai X (2018) Description of the last-instar larva and pupa of a leaf-mining hispine – *Prionispachampaka* Maulik, 1919 (Coleoptera, Chrysomelidae, Cassidinae, Oncocephalini). ZooKeys 726: 47–60. 10.3897/zookeys.726.21041 PMC5799732 29416391

[B12] Maulik S (1932) On the structure of larvae of hispine beetles-II. Proceedings of the Zoological Society of London 192: 293–322.

[B13] Medvedev LN (1957) Hispid beetles (Coleoptera, Chrysomelidae, Hispinae) of the fauna of the USSR. Zoologischesky Zhurnal 36: 293–296. [In Russian]

[B14] Medvedev LN (1968) On larvae of Hispinae (Coleoptera, Chrysomelidae) of the fauna of the USSR. Zoologischesky Zhurnal 47: 79–84. [In Russian]

[B15] Paulian R (1949) Recherches sur les insectes d’importance biologique de Madagascar I. Mémoires de l’Institut Scientifique de Madagascar 3(A): 348–391.

[B16] Staines CL (2015) Tribe Hispini Catalog of the hispines of the World (Coleoptera: Chrysomelidae: Cassidinae). http://entomology.si.edu/Collections_Coleoptera-Hispines.html [Accessed 14 September 2017]

[B17] Świętojańska J Chorzępa M Ghate H (2006) Description of last instar larva and pupa of *Chaeridionapicea* Baly, 1869 and *Oncocephalaquadrilobata* (Guérin, 1844) (Coleoptera: Chrysomelidae: Cassidinae: Oncocephalini) from India. Zootaxa 1341: 49–68. 10.11646/zootaxa.1341.1.3

[B18] Świętojańska J Kovac D (2007) Description of immatures and the bionomy of the Oriental leaf beetle *Chaeridionathailandica* Kimoto, 1998 (Coleoptera: Chrysomelidae: Cassidinae: Oncocephalini), a leaf-mining hispine beetle. Zootaxa 1637: 21–36. 10.11646/zootaxa.1637.1.2

[B19] Świętojańska J Borowiec L Stach M (2014) Redescription of immatures and bionomy of the Palaearctic species *Dicladispatestacea* (Linnaeus, 1767) (Coleoptera: Chrysomelidae: Cassidinae: Hispini), a leaf-mining hispine beetle. Zootaxa 3811(1): 1–33. 10.11646/zootaxa.3811.1.1 24943146

[B20] T’an J (1993) Coleoptera: Hispidae– Hispinae. In: Huang C-M (Ed.) Animals of Longqi Mountain.The series of the bioresources expedition of the Longqi Mountain Nature Reserve, 380–383. [In Chinese]

[B21] Uhmann E (1956) Hispinae aus Indonesia. 170. Beitrag zur Kenntnis der Hispinae (Coleoptera, Chrysomelidae). Beaufortia, Series of Miscellaneous Publications 5(50): 61–72.

[B22] Uhmann E (1957) Hispinae aus dem Britischen Museum. IX. Teil. 184. Beitrag zur Kenntnis der Hispinae (Coleopt. Chrysomelidae). The Annals and Magazine of Natural History (12)10: 364–368. 10.1080/00222935708655969

[B23] Uhmann E (1962) *Dactylispacapicola* (Péringuey) und Verwandte. (203. Beitrag zur Kenntnis der Hispinae (Coleoptera, Chrysomelidae)). Annals of the South African Museum 46(8): 223–230.

[B24] Yano T (1965) Larval stages of the leaf-miners found in Shikoku (Coleopterous leaf-miners of Japan, VII). Transactions of the Shikoju Entomological Society, Marsuyama, 8(4): 115–132.

[B25] Zaitsev YM (2012) The immature stages of the leaf-beetle genus *Dactylispa* (Coleoptera, Chrysomelidae) from Vietnam. Entomological Review 92(3): 305–314. http://dx.doi. org/10.1134/S0013873812030074

